# MCL-1 Inhibition Overcomes Anti-apoptotic Adaptation to Targeted Therapies in B-Cell Precursor Acute Lymphoblastic Leukemia

**DOI:** 10.3389/fcell.2021.695225

**Published:** 2021-09-09

**Authors:** Albert Manzano-Muñoz, Clara Alcon, Pablo Menéndez, Manuel Ramírez, Felix Seyfried, Klaus-Michael Debatin, Lüder H. Meyer, Josep Samitier, Joan Montero

**Affiliations:** ^1^Institute for Bioengineering of Catalonia (IBEC), Barcelona Institute of Science and Technology (BIST), Barcelona, Spain; ^2^Stem Cell Biology, Developmental Leukemia and Immunotherapy, Josep Carreras Leukemia Research Institute-Campus Clinic, Department of Biomedicine, School of Medicine, University of Barcelona, Barcelona, Spain; ^3^Centro de Investigación Biomédica en Red de Cáncer (CIBERONC), Instituto de Salud Carlos III (ISCIII), Institució Catalana de Recerca i Estudis Avançats (ICREA), Barcelona, Spain; ^4^Department of Pediatric Hematology and Oncology, Niño Jesús University Children’s Hospital, Madrid, Spain; ^5^Department of Pediatrics and Adolescent Medicine, Ulm University Medical Center, Ulm, Germany; ^6^Department of Electronics and Biomedical Engineering, Faculty of Physics, University of Barcelona, Barcelona, Spain; ^7^Networking Biomedical Research Center in Bioengineering, Biomaterials and Nanomedicine (CIBER-BBN), Madrid, Spain

**Keywords:** pediatric leukemia, targeted therapies, resistance, apoptosis, BH3 mimetics

## Abstract

Multiple targeted therapies are currently explored for pediatric and young adult B-cell precursor acute lymphoblastic leukemia (BCP-ALL) treatment. However, this new armamentarium of therapies faces an old problem: choosing the right treatment for each patient. The lack of predictive biomarkers is particularly worrying for pediatric patients since it impairs the implementation of new treatments in the clinic. In this study, we used the functional assay dynamic BH3 profiling (DBP) to evaluate two new treatments for BCP-ALL that could improve clinical outcome, especially for relapsed patients. We found that the MEK inhibitor trametinib and the multi-target tyrosine kinase inhibitor sunitinib exquisitely increased apoptotic priming in an NRAS-mutant and in a *KMT2A*-rearranged cell line presenting a high expression of FLT3, respectively. Following these observations, we sought to study potential adaptations to these treatments. Indeed, we identified with DBP anti-apoptotic changes in the BCL-2 family after treatment, particularly involving MCL-1 – a pro-survival strategy previously observed in adult cancers. To overcome this adaptation, we employed the BH3 mimetic S63845, a specific MCL-1 inhibitor, and evaluated its sequential addition to both kinase inhibitors to overcome resistance. We observed that the metronomic combination of both drugs with S63845 was synergistic and showed an increased efficacy compared to single agents. Similar observations were made in BCP-ALL *KMT2A*-rearranged PDX cells in response to sunitinib, showing an analogous DBP profile to the SEM cell line. These findings demonstrate that rational sequences of targeted agents with BH3 mimetics, now extensively explored in clinical trials, may improve treatment effectiveness by overcoming anti-apoptotic adaptations in BCP-ALL.

## Introduction

Acute lymphoblastic leukemia (ALL) is characterized by uncontrolled growth of lymphoid cells and it accounts for 25% of all pediatric cancers (Howlader et al., 2015). Three out of four cases of pediatric ALL are caused by B-cell precursors, also named BCP-ALL (Schwab and Harrison, 2018). BCP-ALL diagnosed patients are typically treated with a chemotherapeutic combination of vincristine, asparaginase, a corticosteroid (prednisone or dexamethasone) and an anthracycline (doxorubicin or daunorubicin), and most patients achieve a complete remission (PDQ Pediatric Treatment Editorial Board, 2002). Despite this outstanding treatment effectiveness, around 15–20% of patients relapse (Locatelli et al., 2012) causing an overall survival rate of 90% (O’Brien et al., 2018). And because of its high incidence this type of tumor is still the deadliest pediatric cancer. Relapsed and refractory patients (R/R) that fail to achieve a complete remission, present highly resistant tumors forcing clinicians to use more aggressive and highly toxic treatments (Oskarsson et al., 2018). Even those patients that are cured face long-term secondary effects including mental problems, functional impairment, cardiotoxicity and increased morbidity (Mody et al., 2008). There is a clear need for new treatments to enhance tumor elimination while reducing lasting toxicity.

Important efforts have been made to identify and characterize oncogenic molecular targets to block them and impair tumor growth and progression. Since the first successes treating pediatric ALL patients using folic acid antagonists achieved by Sydney Farber in the late 1940s (Miller, 2006), a myriad of compounds targeting multiple proteins have been explored in clinical trials. The first and most successful targeted therapy approved for hematological malignancies was imatinib for adult chronic myelogenous leukemia (Deininger, 2008). Interestingly, imatinib targets the BCR-ABL fusion protein that is also present in the Philadelphia chromosome-positive (Ph +) B-cell acute lymphoblastic leukemia (B-ALL) subtype. Clinical trials evaluating imatinib and dasatinib for pediatric and young adult Ph + B-ALL showed improvement in treatment response (Biondi et al., 2012; Gore et al., 2018). Besides BCR-ABL fusion protein, JAK/STAT pathway proteins, FLT3 receptor, MAPK pathway proteins, precursor-B-cell receptor (pre-BCR) or the ubiquitin-proteasome system have been proposed as potential targets for different B-ALL subtypes, increasing the armamentarium of potential targeted therapies for this disease (Kuhlen et al., 2019).

Identifying new effective treatments for pediatric cancer is challenging. If these therapies are not correctly assigned, there is a risk to provoke undesired secondary effects. Precision medicine aims to correctly assign effective treatments to every patient based on molecular characterization of the tumor (Jameson and Longo, 2015). Its successful implementation will lead to more effective therapeutic regimes and reduce side effects (Mathur and Sutton, 2017). Genetic analyses are the most common strategy used to identify cancer patients that would benefit from targeted therapies (Malone et al., 2020). However, the pediatric population presents a low number of mutations compared to adult population, making difficult to identify predictive biomarkers (Savary et al., 2020). In contrast, functional assays overcome these drawbacks by directly exposing patient-isolated tumor cells to different treatment options and measuring their effectiveness (Howard et al., 2017). Yet, this approach presents a major limitation: primary cells rapidly decay, lose their viability and experience phenotypic changes, preventing their use in prolonged assays (Meijer et al., 2017). In this regard, the functional assay dynamic BH3 profiling (DBP) (Montero et al., 2015) can identify effective anti-cancer treatments in less than 24 h directly on patient samples.

Most anti-cancer agents engage cell death by apoptosis, a process regulated by the BCL-2 family of proteins. Inside this family, BAX and BAK are considered effector members and once activated, oligomerize to form pores and induce the Mitochondrial Outer Membrane Permeabilization (MOMP) which represents the point of no return for the apoptotic process (Wei et al., 2001) and the cell commitment to die (Letai, 2011). MOMP induces the release of cytochrome c (and other proteins) from the mitochondria into the cytosol, and its binding to APAF-1 and caspase-9 form the apoptosome, which activates downstream effector caspases and executes apoptosis. Effector proteins are activated by proteins presenting a unique BCL-2 homology (BH) domain, known as BH3-only activator proteins (BIM, BID, and PUMA). The anti-apoptotic BCL-2 family proteins (BCL-2, BCL-xL, MCL-1, BCL-w, and BFL-1) can inhibit both effectors and activator members, hence protecting cells from apoptosis. A fourth subgroup of BCL-2 family proteins, the sensitizers – that include BAD, HRK and NOXA among others – exert a pro-apoptotic effect by specifically inhibiting the anti-apoptotic proteins (Letai et al., 2002).

Dynamic BH3 profiling uses synthetic peptides that mimic the BH3 domain of the pro-apoptotic BH3-only, with a similar effect as the full-length protein. It uses these peptides to measure how close cells are to commit apoptosis, or how “primed” are for death, after a short incubation with the treatment. DBP has been extensively used to identify effective anti-cancer treatments in cell lines, patient-derived xenografts (PDX) and directly on patient-isolated cells from different types of cancer (Montero et al., 2015, 2017, 2019; Wu et al., 2015; Townsend et al., 2016; Deng et al., 2017; Alcon et al., 2020) with an excellent predictive capacity. Interestingly, using sensitizer-analog BH3 peptides, DBP can identify the anti-apoptotic protein used by cancer cells to survive therapy (Frenzel et al., 2009; Montero and Letai, 2018). In fact, most cytogenetic abnormalities found in BCP-ALL regulate anti-apoptotic BCL-2 proteins. For example, the BCR-ABL fusion protein upregulates BCL-2 and ETV6/RUNX1 promotes BCL-xL overexpression (Brown et al., 2017). Hereof, BH3 mimetics, anti-apoptotic inhibitors widely explored in clinical trials, can be used to overcome this therapy-acquired resistance. In fact, it has been demonstrated in ALL that combining standard-of-care treatment with BH3 mimetics can greatly increase efficacy (Ni Chonghaile et al., 2014; Iacovelli et al., 2015; Khaw et al., 2016; Seyfried et al., 2019). But the key question remains unsolved: how and when to better utilize them in the clinic.

Here, we use DBP to identify new effective therapeutic strategies for pediatric and adolescent BCP-ALL. We tested two promising targeted therapies, trametinib (MEK inhibitor) and sunitinib (multi-target tyrosine kinase inhibitor), currently explored in pre-clinical studies. We found that both caused an MCL-1 dependence to protect BCP-ALL cells from apoptotic cell death and that its inhibition with a BH3 mimetic significantly enhanced leukemia cell death. Finally, we were able to observe similar anti-apoptotic adaptations in a pediatric BCP-ALL PDX, demonstrating its potential use in the clinic.

## Materials and Methods

### Cell Lines and Treatments

NALM-6 and SEM cell lines were kindly provided by Prof. PM laboratory at the Josep Carreras Leukaemia Research Institute. Both cell lines were cultured in RPMI 1640 medium (31870, Thermo Fisher, Gibco, Paisley, Scotland) with 10% of heat-inactivated fetal bovine serum (10270, Thermo Fisher, Gibco), 1% of L-glutamine (25030, Thermo Fisher, Gibco) and 1% of penicillin/streptomycin (15140, Thermo Fisher, Gibco). Cells were maintained inside a humidified incubator at 37°C and 5% of CO_2_. Imatinib, dasatinib, sunitinib, trametinib, ibrutinib and ruxolitinib were purchased at SelleckChem (Munich, Germany) and ABT-199, A-1331852 and S63845 were purchased at MedChemExpress (Monmouth Junction, NJ, United States). These reagents were diluted in dimethyl sulfoxide (DMSO) (D8418, Sigma-Aldrich, Saint Louis, MO, United States) and added to the culture media at the indicated concentration and incubation time for every experiment.

### Cell Death Assay

After treatment, cells were resuspended in staining buffer (100 mM HEPES free acid, 40 mM KCl, 1.4 M NaCl, 7.5 mM MgCl_2_ and 25 mM CaCl_2_ at pH 7.4) with Alexa Fluor 647^®^ conjugated Annexin V (640912, BioLegend) and DAPI (62248, Thermo Fisher). Cells were analyzed with a Gallios flow cytometer (Beckman Coulter, Nyon, Switzerland) and results were analyzed with FlowJo software to quantify viable cells (Annexin V and DAPI negative events). Results were represented as the mean of %Cell death (100-%viable cells) of at least three independent experiments.

### Dynamic BH3 Profiling

Dynamic BH3 profiling (DBP) was performed as previously described (Ryan et al., 2016; Montero et al., 2019; Alcon et al., 2020). After treatment, cells were stained using the viability marker Zombie Violet (423113, BioLegend, Koblenz, Germany) for 10 min at room temperature, washed with PBS and resuspended in 330 μL of MEB buffer (150 nM mannitol, 10 mM HEPES-KOH pH 7.5, 150 mM KCl, 1 mM EGTA, 1 mM EDTA, 0.1% BSA and 5 mM succinate). In parallel, peptide solutions were prepared using MEB buffer with 0.002% of digitonin (D141, Sigma-Aldrich) and 12 different peptide solutions with final concentrations of 25 μM of alamethicin (BML-A150-0005, Enzo Life Sciences, Lörrach, Germany), 10 μM, 3 μM, 1 μM, 0.3 μM, 0.1 μM, 0.03 μM, and 0.01 μM of BIM BH3 peptide, 0.1 μM of BAD BH3 peptide, 100 μM of HRK BH3 peptide, 10 μM of MS1 BH3 peptide and a DMSO only control. A 25 μL of cells was added to 25 μL of each peptide solution in a 96-well plate (3795, Corning, Madrid, Spain) and incubated at room temperature for 1 h. After this incubation, cells were fixed with 25 μL of an 8% paraformaldehyde solution for 15 min and neutralized with 50 μL of N2 buffer (1.7 M tris base, 1.25 M glycine at pH 9.1). Finally, 25 μL of intracellular staining buffer (1% Tween20, 5% BSA in PBS) with 1:1,000 dilution of the cytochrome C antibody (Alexa Fluor^®^ 647 anti-Cytochrome c—6H2.B4, 612310, BioLegend) was added and plates were incubated overnight at 4°C. Results were analyzed using a Sony flow cytometer (SONY SA3800, Surrey, United Kingdom) and processed with FlowJo to quantify cytochrome c release (%priming). Δ%priming stands for the difference of %priming between non-treated cells and treated cells for every specific peptide. All results are represented as the mean of at least three independent experiments.

### Protein Extraction

After treatment, cells were collected and washed with PBS. Then, cells were resuspended in RIPA buffer [150 mM NaCl, 5 mM EDTA, 50 mM Tris–HCl pH = 8, 1% Triton X-100, 0.1% SDS, EDTA-free Protease Inhibitor Cocktail (4693159001, Roche, MannKind, Germany)] and lysed for 30 min on ice following centrifugation at 4°C for 10 min at 16,000*g*. Protein in the supernatant was quantified using Pierce^TM^ BCA Protein Assay Kit (23227, Thermo Fisher) and stored at −20°C.

### Immunoprecipitations

Lysates were obtained following the protein extraction protocol but using immunoprecipitation (IP) lysis buffer (150 mM NaCl, 10 mM Hepes, 2 mM EDTA, 1% Triton X-100, 1.5 mM MgCl_2_, 10% glycerol and EDTA-free Protease Inhibitor Cocktail (4693159001, Roche, MannKind, Germany). Again, the protein extracted was quantified with Pierce^TM^ BCA Protein Assay Kit and stored at −20°C. Equivalent amount of protein was incubated at 4°C overnight with magnetic beads (161-4021, Bio-Rad, Madrid, Spain) previously conjugated to 5 μg of rabbit anti-MCL-1 antibody (CST94296, Cell Signaling, Leiden, Netherlands) or 5 μg of rabbit-IgG antibody (CST2729, Cell Signaling). A 30 μL of protein for each condition was stored at −20°C as the input fraction. After incubation, tubes were magnetized to obtain the binding fraction. The supernatant was extracted and stored at −20°C as the unbound fraction. The binding fraction was cleaned with PBS-T (PBS with 0.1% Tween 20) and resuspended in 40 μL of 4× Laemmli sample buffer (161-0747, Bio-Rad), then heated at 70°C to allow separation between the target protein and the magnetic beads-antibody complex. The sample was magnetized again and the supernatant containing the pulled-down proteins was stored at −20°C as IP fractions.

### Immunoblotting

An equal amount of protein was prepared in 4× Laemmli buffer (161-0747, Bio-Rad) and heated at 96°C for 10 min. The sample was loaded in an SDS-PAGE gel (456-1025, Bio-Rad) and proteins were separated for 2 h. Then, proteins were transferred to a PVDF membrane (10600023, Amersham Hybond, Pittsburgh, PA, United States) at 55V for 2 h at 4°C. The membrane was blocked with 5% dry milk in TBS-T (50 mM Tris–HCl pH = 7.5, 150 mM NaCl and 1% Tween20) for 1 h and washed with TBS-T. After blocking, the membrane was probed overnight at 4°C in TBS-T with 3%BSA and 1:1,000 dilution of the primary antibody against the protein of interest: rabbit anti-BCL-2 (CST4223, Cell Signaling), rabbit anti-BCL-xL (CST2764, Cell Signaling), rabbit anti-MCL-1 (CST5453, Cell Signaling), rabbit anti-BIM (CST2933, Cell Signaling), rabbit anti-BAX (CST2772, Cell signaling), rabbit anti-BAK (CST12105, Cell signaling), phospho-p44/42 MAPK (Erk1/2) (Thr202/Thr204) (CST4370, Cell signaling), phospho-BIM (Ser69) (CST4585, Cell signaling) and rabbit anti-Actin (CST4970, Cell Signaling) followed by 1 h at room temperature with 1:3,000 of anti-rabbit IgG HRP-linked secondary antibody (CST7074, Cell Signaling). When necessary, membranes were stripped using mild stripping buffer (0.1 M glycine pH 2.5 and 2% SDS) for two cycles of 20 min at 50°C and extensively washed with TBS (50 mM Tris–HCl pH = 7.5 and 150 mM NaCl). Membranes were developed using Clarity ECL Western substrate (1705060, Bio-Rad) in a LAS4000 imager (GE Healthcare Bio-Sciences AB, Uppsala, Sweden). Bands were quantified using ImageJ software to measure the integrated optical density.

### Precursor BCP-ALL PDX Model Generation

The primary leukemia specimen was obtained from peripheral blood of an infant patient with newly diagnosed pro-B ALL after informed written consent with the legal guardians and in accordance with the institution’s ethical review board. Xenograft cells were established by intravenous injection of ALL cells into female NOD/SCID mice (NOD.CB17-Prkdcscid, Charles River) as described earlier (Meyer et al., 2011; Seyfried et al., 2019). Animal experiments were approved by the appropriate authority (Tierversuch Nr. 1260, Regierungspräsidium Tübingen).

### DBP With PDX Cells

After treatment, cells were stained using the viability marker Zombie Violet (423113, BioLegend, Koblenz, Germany) for 10 min on ice, followed by staining for 30 min on ice with 1:200 dilution of Alexa Fluor^®^ 488 anti-human CD45 antibody (368536, BioLegend) and PE anti-human CD19 antibody (392506, BioLegend) in TBS with 10%FBS. Then, cells were washed with PBS and resuspended in 330 μL of MEB buffer. After this, DBP was done as explained in point 2.3.

### Statistical Analysis

For the ROC curve analysis, data sets were separated as responders (cell death >10%) and non-responders (cell death <10%) and their corresponding values of Δ%priming were used to perform the analysis. Synergies were calculated using the Bliss Independent model (Foucquier and Guedj, 2015) were the Combination Index (CI) is calculated as CI = (CD_A_ + CD_B_ – CD_A_^∗^CD_B_)/CD_combi__nation_ (CD stands for the percentage of cell death after treatment A, B or the combination of them). Combinations with CI < 1 were considered synergistic. GraphPad Prism 9 was used to perform statistical analyses and generate graphs.

## Results

### DBP Predicts Cytotoxicity in BCP-ALL Cell Lines

Targeted therapies are currently explored in clinical trials to treat pediatric and young adult BCP-ALL. For example, imatinib and dasatinib for patients presenting the BCR-ABL fusion protein (Ph + cases); trametinib for RAS-mutant patients (Jerchel et al., 2018); sunitinib for cases with overexpression or activating mutations of FLT3 (Brown et al., 2005); ruxolitinib for tumors with constitutive activation of the JAK/STAT signaling pathway (Ding et al., 2018), and ibrutinib when pre-BCR is active (Kim et al., 2017). We sought to explore the effects of these targeted therapies, particularly on the apoptotic pathway, in BCP-ALL.

**FIGURE 1:**
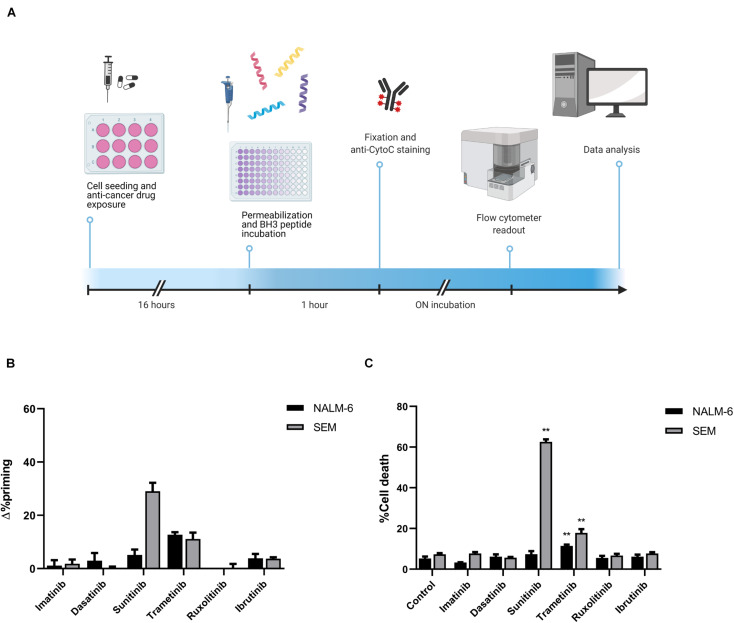
Cytotoxicity induced by targeted drugs in BCP-ALL cell lines. **(A)** Graphical scheme of the DBP technique where cancer cells are plated and treated for 16 h. After drug exposure, cells are plated in 96-well plates and exposed to the different BH3 peptides. After 1 h, cells were fixed and stained with an anti-Cytochrome C antibody overnight. Finally, analyses were performed using a flow cytometer for drug-response curves. Designed with BioRender.com. **(B)** DBP with BIM BH3 peptide after 16 h of incubation with 1,000 nM imatinib, 100 nM dasatinib, 1,000 nM sunitinib, 100 nM trametinib, 100 nM ruxolitinib, and 1,000 nM ibrutinib in NALM-6 and SEM cell lines. Δ%priming stands for the difference in %priming between treatment and control conditions. **(C)** Cytotoxicity expressed as percentage of dead cells after 72 h of treatment with the same therapies assessed by Annexin V/DAPI staining. All results are expressed as the mean ± standard error of the mean (SEM) of at least three biologically independent replicates. Statistical significance was calculated using Student’s *t*-test comparing to control condition and considering *p < 0.05 and **p < 0.01.

To explore the pro-apoptotic effect of these therapies, we performed DBP in two pediatric and young adult cell lines, SEM (presenting *KMT2A*-rearrangement and high expression of FLT3) and NALM-6 (NRAS-mutant), respectively (Figure 1A). After a short incubation with treatments, we observed that sunitinib induced a high increment in apoptotic priming when exposed to the BIM peptide in the SEM cell line, while trametinib increased it mildly in both cell lines. In contrast, imatinib, dasatinib, ruxolitinib and ibrutinib did not produce any induction of apoptosis (Figure 1B). To validate DBP’s predictions, we treated these cells for longer timepoints with the same therapies and assessed cell death induction. When comparing Δ%priming and cytotoxicity, similarly to DBP’s predictions, we observed that sunitinib in the SEM cell line and trametinib in both cell lines were more efficient than the rest of the therapies inducing cell death (Figure 1C). The receiver operating characteristic curve analysis was then used to assess the predictive capacity of DBP on identifying cytotoxic treatments. Our results showed an area under the curve of 1, confirming that DBP is an excellent predictor for this experimental subset (Supplementary Figure 1A). Furthermore, Δ%priming strongly correlated with cell death after 3 days of treatment, suggesting that a higher increase in apoptotic priming is an early predictor for cytotoxicity in these cells (Supplementary Figure 1B). Altogether, these results demonstrate that DBP could be used as a predictive biomarker to find effective therapies for BCP-ALL.

### Trametinib Induces MCL-1 Dependence in NALM-6 Cell Line

**FIGURE 2:**
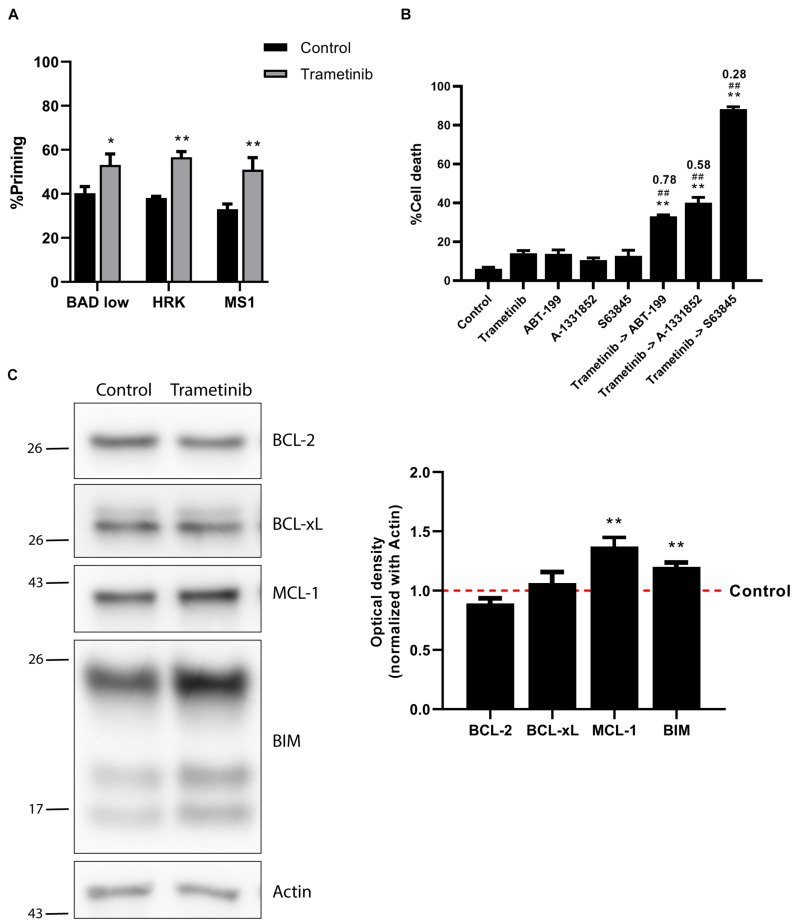
Trametinib synergizes with S63845 in NALM-6 cells, increasing BIM and MCL-1 protein expression. **(A)** DBP results using sensitizer peptides to study anti-apoptotic dependence of BCL-2 and BCL-xL with BAD 0.1 μM, BCL-xL with HRK 100 μM and MCL-1 with MS1 10 μM after 16 h of incubation with trametinib 100 nM in NALM-6 cell line. **(B)** Cytotoxicity was assessed by Annexin V/DAPI staining after 96 h incubation with trametinib 100 nM, ABT-199 100 nM, A-1331852 100 nM and S63845 1,000 nM in NALM-6 cell line. BH3 mimetics in combination with trametinib were added 16 h after treatment. **(C)** Western blot analysis for anti-apoptotic and BIM proteins in NALM-6 cells after 16 h of treatment with trametinib 100 nM. Quantification of optical density for each protein was normalized to actin, and fold-change was calculated comparing to protein expression in the control condition. All results are expressed as the mean ± standard error of the mean (SEM) of at least three biologically independent replicates. Statistical significance was calculated using Student’s *t*-test compared to control condition and considering *p < 0.05 and **p < 0.01. Significance was also calculated comparing combination conditions with both single agents and considering #*p* < 0.05 and ##*p* < 0.01. Combination Index (CI) is indicated on top of every combination where CI < 1 indicates synergy.

As previously mentioned, trametinib was the only targeted therapy tested that showed some efficacy in NALM-6 cells. However, only 20% of the cells were dead after 72 h of treatment, showing a modest efficacy. Multiple studies previously reported cancer cells’ adaptation to therapy and the key role of the anti-apoptotic BCL-2 family proteins in their resistance to cell death (Reed et al., 1996; Mansoori et al., 2017; Maji et al., 2018; Wei et al., 2020). We next sought to study the role of these proteins after trametinib treatment on NALM-6. We repeated the DBP analyses, but instead of using the BIM BH3 peptide, we used peptides mimicking the sensitizer members of the BCL-2 family to specifically identify the anti-apoptotic proteins’ contribution. In this case, we used a low concentration of the BAD BH3 peptide, due to the exquisite sensitivity of both BCP-ALL cell lines to this peptide. In brief, an increase in apoptotic priming after incubation with BAD BH3 peptide would mean that BCL-2 and perhaps BCL-xL are involved in cell resistance to trametinib. Similarly, a gain in apoptotic priming with HRK BH3 would indicate an enhanced BCL-xL contribution, while an MS1 BH3 signal increase would point to MCL-1. When we analyzed NALM-6 treated with trametinib, we observed a positive signal from all sensitizer peptides (Figure 2A), suggesting that multiple anti-apoptotic proteins may be involved in cell survival after therapy. We next studied how to overcome this acquired resistance by preincubating the cells with trametinib and then adding specific BH3 mimetics – small molecules that specifically block anti-apoptotic proteins. When we sequentially combined trametinib with the BCL-2 inhibitor ABT-199 (venetoclax), the BCL-xL inhibitor A-1331852 or the MCL-1 inhibitor S63845, we observed enhanced cytotoxicity in all the combinations tested compared to single agent treatment. However, the dual inhibition of MEK and MCL-1 was significantly more effective and clearly synergistic (CI = 0.28), achieving almost a complete elimination of these cells (Figure 2B).

**FIGURE 3:**
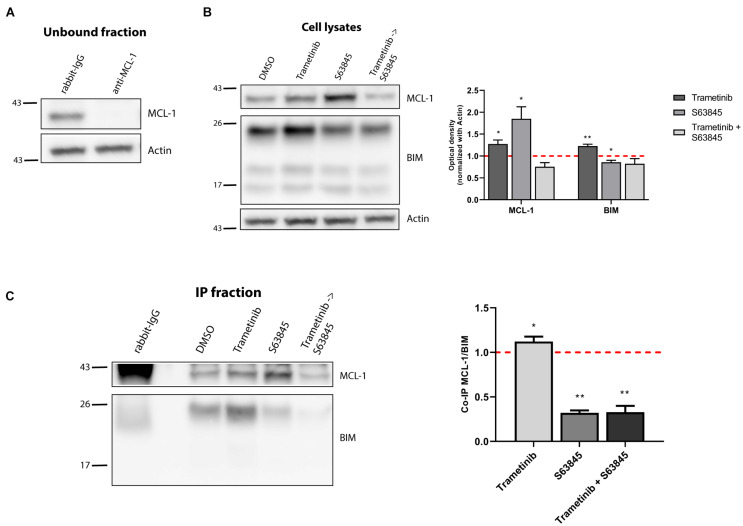
Synergy of trametinib and S63845 in NALM-6 is explained by an increased interaction between MCL-1 and BIM. **(A)** Western blot analysis of the unbound fraction after MCL-1 immunoprecipitation. **(B)** Immunodetection of MCL-1 and BIM initial expression in cell lysates after 16 h of incubation with trametinib 100 nM, and 2 h of incubation with S63845 1,000 nM in the specified conditions. Quantification of optical density for each protein was normalized to actin and fold-change was calculated comparing to protein expression in the control condition. **(C)** Western blot of the immunoprecipitated fraction was used to study the interaction between MCL-1 and BIM after treatment with trametinib 100 nM and 2 h with S63845 1,000 nM. To quantify this binding, BIM optical density was normalized to MCL-1 optical density and fold-change was calculated comparing to protein expression in the control condition. All results are expressed as the mean ± SEM of at least three biologically independent replicates. Statistical significance was calculated using Student’s *t*-test compared to control condition and considering *p < 0.05 and **p < 0.01.

We next examined the molecular mechanism behind the strong trametinib and S63845 synergy. The BCL-2 family of proteins is a complex interactome, thus explaining the observed effectiveness could be challenging. It is well reported that trametinib treatment leads to an increase of the activator BIM, which was also detected in the NALM-6 cell line (Figure 2C). MEK inhibition using trametinib caused a reduction of ERK1/2 phosphorylation, that also reduced BIM phosphorylation (Supplementary Figure 2A), and avoided its proteasomal degradation (O’Reilly et al., 2009). This increase in BIM causes priming for apoptosis, yet the anti-apoptotic members of the BCL-2 family could sequester this protein and block the initiation of apoptosis. When we analyzed the anti-apoptotic proteins expression, we found that this MEK inhibitor selectively promoted MCL-1 increase, while BCL-2 and BCL-xL levels remained unchanged (Figure 2C). In the case of the effector proteins, BAX was not detected in this cell line and BAK slightly increased after trametinib treatment (Supplementary Figure 2B). To confirm that MCL-1 is the main protein binding to BIM, we decided to immunoprecipitate it and study their interaction. MCL-1 was detected in the normal rabbit-IgG unbound fraction but not in the anti-MCL-1 antibody condition (Figure 3A), confirming that we were able to effectively pull it down. As previously observed, trametinib treatment caused an increased binding of MCL-1 with BIM, and S63845 caused a marked increase in MCL-1 (Figure 3B) due to protein stabilization, as described elsewhere (Kotschy et al., 2016; Li et al., 2019; Montero et al., 2019). As expected, the interaction between MCL-1 and BIM in the IP fraction increased when treated with trametinib compared to control, and was almost completely displaced when S63845 was sequentially added (Figure 3C). These findings confirm MCL-1 as the main pro-survival protein and correlate with the observed synergy (Figure 2B).

MCL-1 preferentially blocks the increase of BIM protein after trametinib exposure, and when sequentially inhibited with the BH3 mimetic S63845, over 80% of the cells succumbed to this synergistic combination (Figure 2B). Interestingly, when we combined trametinib with ABT-199 or A-1331852 only a modest cytotoxic effect was observed (Figure 2B). Based on these findings, we conclude that MCL-1 is the main anti-apoptotic protein against trametinib-induced apoptosis in NALM-6, and that BCL-2 and BCL-xL play a minor role.

### Combination of Low-Dose Sunitinib and BH3 Mimetics Synergize to Maintain Treatment Efficacy

**FIGURE 4:**
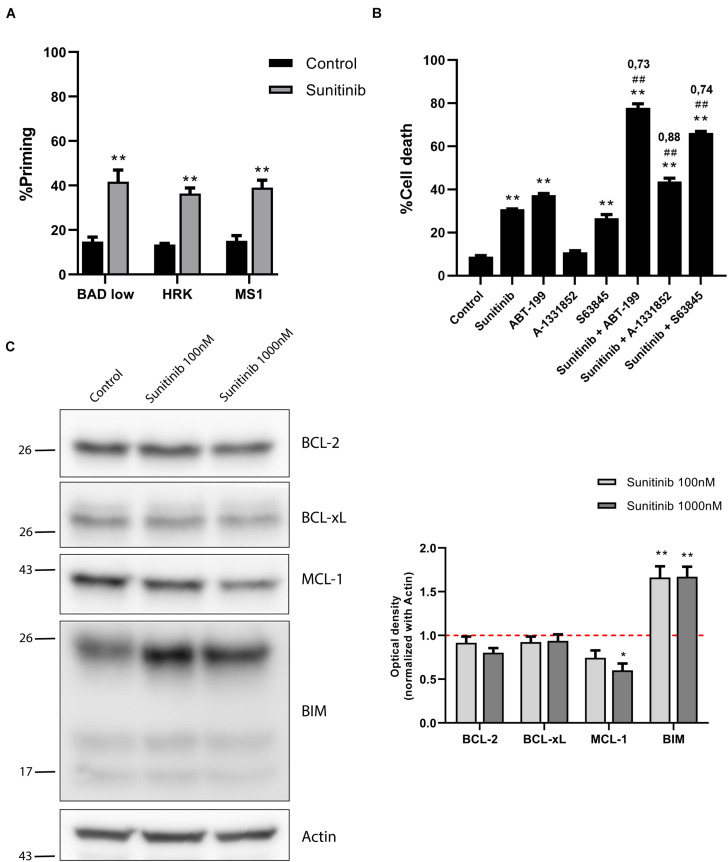
Sunitinib synergizes with ABT-199 and S63845 in SEM cells but anti-apoptotic proteins are downregulated. **(A)** DBP results using sensitizers peptides to study anti-apoptotic dependence of BCL-2 and BCL-xL with BAD 0.1 μM, BCL-xL with HRK 100 μM and MCL-1 with MS1 10 μM after 16 h of incubation with sunitinib 1,000 nM in SEM cell line. **(B)** Cytotoxicity assessed by Annexin V/DAPI staining after 96 h of sunitinib 100 nM, ABT-199 10 nM, A-1331852 100 nM and S63845 100 nM exposure in SEM cells. BH3 mimetics in combination with sunitinib were added 16 h after treatment. **(C)** Western blot analysis for anti-apoptotic and BIM proteins in SEM cells after 16 h of treatment with sunitinib 100 nM and sunitinib 1,000 nM. Quantification of optical density for each protein was normalized to actin, and fold-change was calculated comparing to protein expression in the control condition. All results are expressed as the mean ± SEM of at least three biologically independent replicates. Statistical significance was calculated using Student’s *t*-test compared to control condition and considering *p < 0.05 and **p < 0.01. Significance was also calculated comparing combination conditions with both single agents and considering #*p* < 0.05 and ##*p* < 0.01. CI is indicated on top of every combination where CI < 1 indicates a synergistic combination.

Secondary effects resulting from anti-cancer therapy are particularly threating for the pediatric population (Sarosiek et al., 2017). As a result, there is a trend to substitute high-dose single agent treatment for low-dose combinations targeting different vulnerabilities to maximize efficacy and reduce toxicity (Satti, 2009). In this regard, we sought to find a combination with BH3 mimetics that could synergize with sunitinib, currently explored in pre-clinical investigations for adult and pediatric BCP-ALL (Brown et al., 2005; Griffith et al., 2016). In the SEM cell line, sunitinib, administered as a single agent, killed around 60% of the cells (Figure 1C), but we aimed to improve its efficacy. By performing DBP analyses, we found that sunitinib incubation, enhanced BAD, HRK and MS1 sensitizer BH3 peptides priming, also suggesting a diversified anti-apoptotic adaptation to this targeted therapy (Figure 4A). We then explored reducing 10-fold the concentration of sunitinib, aiming to diminish the potential secondary effects in the clinic, and combining with BH3 mimetics to boost its anti-cancer efficacy. Two BH3 mimetics, ABT-199 and S63845, also induced high levels of cytotoxicity as single agents (Supplementary Figure 3), but were innocuous when lowering 10-fold their concentration (Figure 4B). To improve the performance of the low-dose sunitinib treatment, we combined it with three BH3 mimetics targeting different anti-apoptotic proteins. As anticipated by DBP, when blocking any of the three anti-apoptotic proteins studied following sunitinib treatment, we found a significant increase in cytotoxicity. This synergistic effect was especially notable when combining it with low-dose ABT-199 (CI = 0.73) or S63845 (CI = 0.74) (Figure 4B).

We hypothesized that the molecular mechanism explaining these combinations could be similar to the one found in NALM-6 after trametinib administration. Similarly, we observed a marked increase in BIM expression after sunitinib treatment (Figure 4C), as previously described for other types of tumors (Yang et al., 2010). Interestingly, sunitinib also reduced ERK1/2 and BIM phosphorylation (Supplementary Figure 4A), suggesting that this inhibitor also affects MAPK pathway, as previously described for other cancer types (Chahal et al., 2010; Fenton et al., 2010). Sunitinib clearly increases apoptotic priming in the SEM cell line; yet, it also activates the anti-apoptotic machinery to neutralize the increase of pro-apoptotic proteins. When we analyzed the BCL-2 family components, in contrast to what we observed in NALM-6, there was a minor decrease in BCL-2 and a significant reduction of MCL-1 expression (Figure 4C). No significant changes were observed for BAX and BAK (Supplementary Figure 4B). Surprisingly, these results seemed to antagonize the observed synergies with ABT-199 or S63845.

**FIGURE 5:**
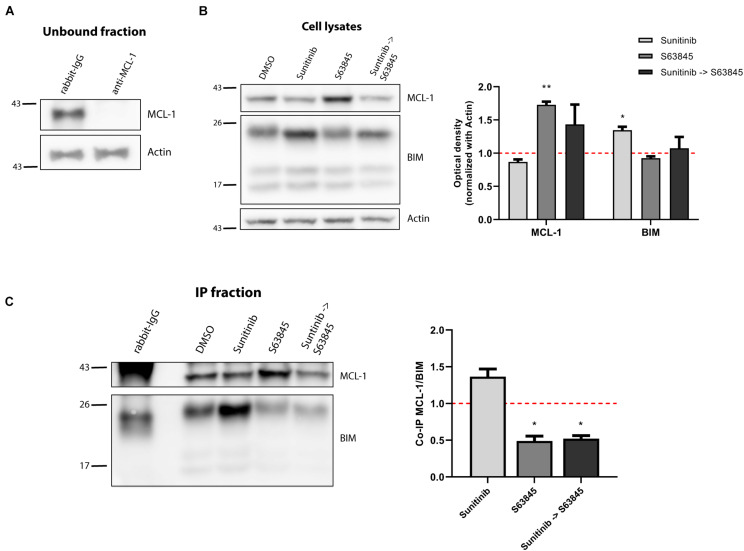
Increased binding of BIM to MCL-1 causes sunitinib and S63845 synergism. **(A)** Western blot analysis of the unbound fraction after MCL-1 immunoprecipitation. **(B)** Immunodetection of MCL-1 and BIM initial expression in cell lysates after 16 h of incubation with sunitinib 100 nM, and 2 h incubation with S63845 100 nM in the specified conditions. Quantification of optical density for each protein was normalized to actin and fold-change was calculated comparing to protein expression in the control condition. **(C)** Western blot of the immunoprecipitated fraction was used to study the interaction between MCL-1 and BIM after treatment with sunitinib 100 nM and 2 h with S63845 100 nM. To quantify this binding, BIM optical density was normalized to MCL-1 optical density and fold-change was calculated comparing to protein expression in the control condition. All results are expressed as the mean ± SEM of at least three biologically independent replicates. Statistical significance was calculated using Student’s *t*-test compared to control condition and considering *p < 0.05 and **p < 0.01.

As already mentioned, when exposed to a perturbation like a cytotoxic agent, cancer cells may adapt using anti-apoptotic proteins. However, in this case, a counterintuitive decrease in these proteins was detected. To further elucidate how SEM cells survive sunitinib, we immunoprecipitated MCL-1 (Figure 5A) to study its interaction with BIM. As expected, cell lysates showed a significant lower expression of MCL-1 and an increase in BIM after sunitinib treatment, and the stabilization of MCL-1 after S63845 (Figure 5B). Interestingly, we observed that sunitinib treatment, even if the overall MCL-1 expression decreased, promoted and increase of its binding to BIM. Then, when MCL-1 was blocked with S63845, BIM was displaced and apoptosis was then restored (Figure 5C). These findings explain why the combination of sunitinib and S63845 synergize. Similarly as before, BCL-2 and BCL-xL could not neutralize BIM after MCL-1 inhibition to prevent cell death.

**FIGURE 6:**
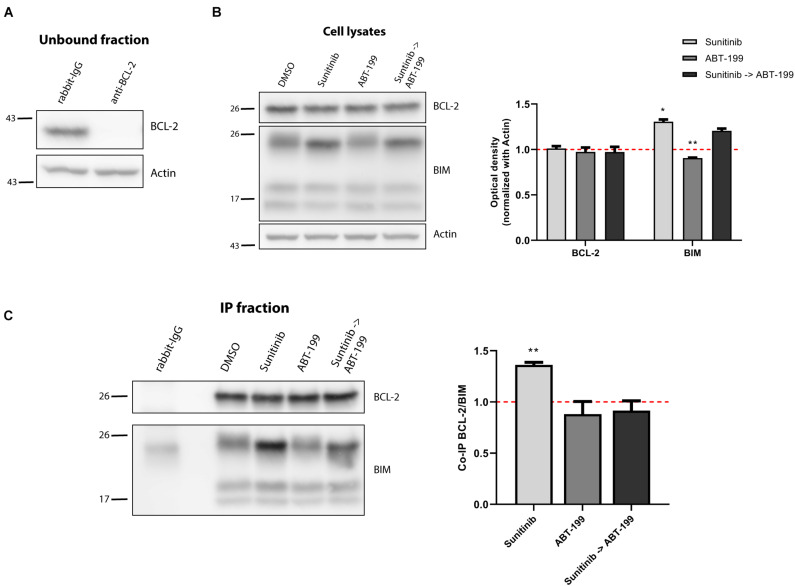
BCL-2 binds to BIM after sunitinib treatment promoting synergy with ABT-199. **(A)** Western blot analysis of the unbound fraction after BCL-2 immunoprecipitation. **(B)** Immunodetection of BCL-2 and BIM initial expression in cell lysates after 16 h of incubation with sunitinib 100 nM, and 4 h incubation with ABT-199 10 nM. Quantification of optical density for each protein was normalized to actin and fold-change was calculated comparing to protein expression in the control condition. **(C)** Western blot of the immunoprecipitated fraction to study the interaction between BCL-2 and BIM after sunitinib 100 nM treatment, and 4 h with ABT-199 10 nM. To quantify this binding, BIM optical density was normalized to BCL-2 optical density and fold-change was calculated comparing to protein expression in the control condition. All results are expressed as the mean ± SEM of at least three biologically independent replicates. Statistical significance was calculated using Student’s *t*-test compared to control condition and considering *p < 0.05 and **p < 0.01.

We aimed to further investigate the other significant synergy detected between sunitinib and the BCL-2 inhibitor ABT-199. In contrast to MCL-1, BCL-2 expression slightly decreased after exposing them to sunitinib (Figure 4C). We followed the previous approach and immunoprecipitated BCL-2, that was effectively pulled down (Figure 6A), and BIM increase was observed after treatment with sunitinib in the cell lysates (Figure 6B). BCL-2 was clearly detected in the immunoprecipitated fraction and we could observe an increased binding between BCL-2 and BIM in the SEM cells when treated; and this interaction was displaced when sequentially administering ABT-199 (Figure 6C). These results suggest that the increase in BIM expression and the induction of apoptosis after low-dose sunitinib treatment is neutralized preferentially by BCL-2 and MCL-1. When sequentially inhibiting BCL-2 or MCL-1 with the corresponding low-dose BH3 mimetic, BIM is then displaced and the start of the apoptotic process is inevitable – the other anti-apoptotic proteins cannot prevent it.

### Pediatric BCP-ALL PDX Recapitulates SEM Anti-apoptotic Adaptation

**FIGURE 7:**
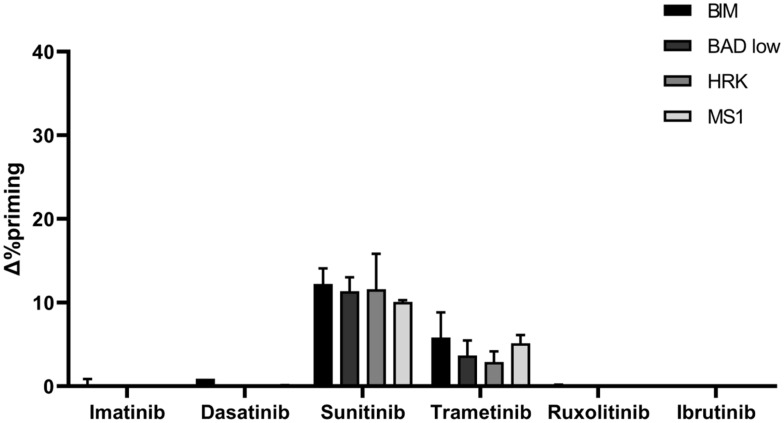
PDX cells presenting *KMT2A* rearrangement show a similar DBP profile as the SEM cells. DBP with BIM BH3, BAD BH3, HRK BH3, and NOXA BH3 peptides after 16 h of incubation with 1,000 nM imatinib, 100 nM dasatinib, 1,000 nM sunitinib, 100 nM trametinib, 100 nM ruxolitinib and 1,000 nM ibrutinib in PDX cells. Δ%priming stands for the difference in %priming between treatment and control condition. All results are expressed as the mean ± standard deviation of two technical replicates.

We next aimed to confirm these anti-apoptotic adaptations using a PDX ALL sample derived from a pediatric BCP-ALL patient with the same *KMT2A*-rearrangement (*KMT2A*/AFF1) present in the SEM cell line (Benito et al., 2015). Following the same experimental conditions, we shortly incubated these cells with the same panel of targeted therapies and studied by DBP the apoptotic induction in the blast population. Similarly as previously described for the cell line, we observed a significant Δ%priming increase with the BIM peptide after sunitinib treatment but a modest increase with trametinib. The other targeted therapies did not produce any perceptible apoptotic changes (Figure 7). Interestingly, we also observed an increase in priming with all three sensitizer peptides (BAD, HRK and MS1) after sunitinib exposure and analogous protein expression changes (Supplementary Figure 5), suggesting similar anti-apoptotic adaptations as the ones observed in SEM cells (Figure 7), which correlates with the akin genetic background.

## Discussion

Pediatric and young adult BCP-ALL patients present an overall survival of 90% (Möricke et al., 2010; Pui et al., 2015). However, the minor subset of R/R cases do worse and display a poor outcome to therapy (Einsiedel et al., 2005; Ko et al., 2010), consolidating ALL as the first cause of death for pediatric cancer. Reinduction therapy for the most advanced cases often include the same treatments previously used but at a much higher concentrations (Raetz and Bhatla, 2012); which is not only ineffective but also increases detrimental secondary effects. There is a clear need for new and effective treatments to improve the survival in those patients that do not respond to actual chemotherapy regimens. Multiple targeted therapies have been proposed as potential candidates to treat different subtypes of BCP-ALL that present genetic targetable vulnerabilities (Kuhlen et al., 2019). Chemotherapy relies on the “one size fits all” concept in which all patients are treated in the same way, but the inclusion of targeted therapies requires a personalized medicine approach, where individual patient tumors are first studied to find a driving oncogene to be exploited pharmacologically. Despite recent advances in genetic screening and current efforts to implement precision medicine to treat pediatric patients (Ginsburg and Phillips, 2018), there is a clear complication: childhood cancers present less genetic alterations compared to adults. More precisely, they present a very low frequency of somatic mutations, thus reducing the number of predictive biomarkers and novel targeted therapies (Gröbner et al., 2018).

The lack of genetic biomarkers is boosting functional assays that directly expose cancer cells to selected potential treatment to measure their efficacy. We and others have demonstrated that DBP is a useful technology to screen and identify effective treatments for many types of cancer, including pediatric (Montero et al., 2015, 2017, 2019; Townsend et al., 2016; Pallis et al., 2017; Grundy et al., 2018; Seyfried et al., 2019; Alcon et al., 2020; Foley, 2020). We performed DBP in two BCP-ALL cell lines by exposing them to potential targeted therapies for this type of leukemia. From all the treatments tested, we identified the MEK inhibitor trametinib as the most effective in the young adult NALM-6 cell line. NALM-6 presents a mutation in NRAS, and downstream activation of the MAPK pathway, that may explain its sensitivity to trametinib (Irving et al., 2014). In another pediatric BCP-ALL cell line, named SEM, DBP predicted trametinib and sunitinib effectiveness that was later confirmed by cell death measurements – the latter was particularly active as single agent. Sunitinib has been proposed as a potential candidate to treat FLT3-driven hematological malignancies (Ikezoe et al., 2006), an alteration that is present in the SEM cell line (Brown et al., 2005; Gu et al., 2011). These results demonstrate DBP’s capacity to functionally identify effective targeted agents without requiring genetic information, which we believe would help personalize R/R BCP-ALL patients treatment.

Since the approval of venetoclax for chronic lymphocytic leukemia, BH3 mimetics have bloomed as potential treatments for multiple types of cancer, predominantly hematological (Valentin et al., 2018). Despite impressive experimental and clinical results as single agents, increasing evidence showed that BH3 mimetics real potential is enhancing other anti-cancer agents, both conventional chemotherapy and targeted (Montero and Letai, 2018; Oudenaarden et al., 2018; Savona and Wei, 2019; Lin et al., 2020). Numerous studies have demonstrated that cancer cells often rely on anti-apoptotic proteins to acquire resistance to therapy, and BH3 mimetics can effectively block these adaptations (Hata et al., 2015; Maji et al., 2018). However, with these new therapeutic strategies we face the same problem described for targeted therapies: when and how to correctly use these BH3 mimetics in the clinic. We previously described that DBP can identify anti-apoptotic adaptations after treatment and guide effective combinations with BH3 mimetics to boost therapy’s potency (Montero et al., 2019; Alcon et al., 2020). When we applied it to BCP-ALL, we found that trametinib as single agent only produced a modest cytotoxic effect on NALM-6 cells, but when sequentially combined with the MCL-1 inhibitor S63845, it reached an almost complete elimination of these cancer cells. Similarly, sunitinib synergized with ABT-199 and S63845 in the SEM cell line, despite reducing 10-fold its concentration. Importantly, all these combinations were anticipated by DBP after only a short incubation with the targeted therapy, demonstrating the utility of this functional assay as predictive biomarker for synergistic combinations of anti-cancer agents with BH3 mimetics.

Anti-apoptotic adaptations in response to therapy may appear by multiple cellular processes. When we analyzed the BCL-2 family of proteins after trametinib and sunitinib treatment, in both cases we observed a significant increase of pro-apoptotic BIM expression, priming cells for apoptosis. Its stabilization after kinase inhibitors use has been previously reported and related to the loss of ERK1/2 phosphorylation (Cragg et al., 2008; Tan et al., 2013; Elgendy et al., 2017), as we confirmed for both agents. Some reports also point out that sunitinib may cause its accumulation by inhibiting STAT3 and AKT (Xin et al., 2009; Yang et al., 2010). Interestingly, changes in anti-apoptotic BCL-2 family members were very different when exposed to both kinase inhibitors. Trametinib increased MCL-1 expression to neutralize BIM, as previously described (Korfi et al., 2016). Although MEK inhibition was reported to enhance BCL-2/xL inhibitors cell death induction in BCP-ALL by Korfi and colleagues, as we also confirmed, as far as we know this is the first time that it is shown that trametinib strongly synergizes with MCL-1 inhibition in this disease. In contrast, sunitinib promoted BIM binding to BCL-2 and MCL-1, despite the overall expression decrease of these pro-survival proteins. Even if the adaptation mechanism was different, we could also overcome it by sequentially adding a BH3 mimetics, venetoclax or S63845, to this targeted agent, demonstrating the therapeutic potential of these molecules. We validated these results using a pediatric BCP-ALL PDX sample obtained from a patient presenting the same *KMT2A* rearrangement as the SEM cell line (Bhimani et al., 2020), detecting similar therapeutic responses. DBP identified sunitinib as the most effective agent inducing apoptotic priming; correlating with the results observed in the SEM cell line, but contrasting to NALM-6 cells that present a different genetic background. Furthermore, we observed similar anti-apoptotic adaptations when exposed to this kinase inhibitor, predicting that low-dose sunitinib combination with ABT-199 or S63845 could enhance the therapeutic effect for this patient. Altogether, these results demonstrate that DBP could be used as companion diagnostic tool to stratify R/R BCP-ALL cases and identify the optimal combination of targeted therapies with BH3 mimetics to maximize anti-cancer efficacy while decreasing undesired secondary effects. Although sunitinib was previously characterized as a potential treatment for BCP-ALL as a FLT3 inhibitor (Griffith et al., 2016), to our knowledge this is the first time that it is described in combination with BH3 mimetics to treat this disease.

In summary, these findings could represent three new potential therapeutic strategies for BCP-ALL. Taking into consideration that venetoclax is already approved for clinical use and that multiple MCL-1 inhibitors are currently explored in clinical trials, we believe that these synergistic combinations that we here describe could likely improve R/R BCP-ALL patient treatment and clinical outcomes.

## Data Availability Statement

The raw data supporting the conclusions of this article will be made available by the authors upon request without undue reservation.

## Ethics Statement

The animal study was reviewed and approved by Tierversuch Nr. 1260, Regierungspräsidium Tübingen.

## Author Contributions

AM-M performed all experiments under supervision of CA and JM. PM provided the cell lines and important advice. FS, K-MD, and LM provided the BCP-ALL PDX cells and related relevant information. MR provided his expertise in the field. JS and JM supervised the work. AM-M and JM wrote the manuscript with the contributions from all authors. All authors contributed to the article and approved the submitted version.

## Conflict of Interest

JM reports previous consulting for Vivid Biosciences and Oncoheroes Biosciences, and his laboratory is currently collaborating with AstraZeneca. The remaining authors declare that the research was conducted in the absence of any commercial or financial relationships that could be construed as a potential conflict of interest.

## Publisher’s Note

All claims expressed in this article are solely those of the authors and do not necessarily represent those of their affiliated organizations, or those of the publisher, the editors and the reviewers. Any product that may be evaluated in this article, or claim that may be made by its manufacturer, is not guaranteed or endorsed by the publisher.

## Supplementary Material

Supplementary Figure 1 **(A)** ROC curve analysis using the values of Δ%priming in NALM-6 and SEM cell lines establishing 10% as the cell death threshold for responders and non-responders. **(B)** Correlation between Δ%priming and %cell death analyses.

Supplementary Figure 2 **(A)** Western blot analysis of phospho-ERK1/2 and phospho-BIM in NALM-6 cell line after 16 h of treatment with trametinib 100 nM. **(B)** Western blot analysis of BAK and BAX in NALM-6 cell line after 16 h of treatment with trametinib 100 nM. Quantification of optical density for each protein was normalized to actin, and fold-change was calculated comparing to protein expression in the control condition. All results are expressed as the mean ± SEM of at least three biologically independent replicates. Statistical significance was calculated using Student’s *t*-test compared to control condition and considering ^∗^*p* < 0.05 and ^∗∗^*p* < 0.01.

Supplementary Figure 3 ABT-199 and S63845 induce cytotoxicity in the SEM cell line. Cytotoxicity expressed as percentage of dead cells after 72 h of treatment with 100 nM ABT-199 and 1,000 nM S63845, as assessed by an Annexin V/DAPI staining. All results are expressed as the mean ± SEM of at least three biologically independent replicates. Statistical significance was calculated using Student’s *t*-test compared to control condition and considering ^∗^*p* < 0.05 and ^∗∗^*p* < 0.01.

Supplementary Figure 4 **(A)** Western blot analysis of phospho-ERK1/2 and phospho-BIM in the SEM cell line after 16 h of treatment with sunitinib 100 nM and 1,000 nM. **(B)** Western blot analysis of BAK and BAX in SEM cell line after 16 h of treatment with sunitinib 100 nM and 1,000 nM. Quantification of optical density for each protein was normalized to actin, and fold-change was calculated comparing to protein expression in the control condition. All results are expressed as the mean ± SEM of at least three biologically independent replicates. Statistical significance was calculated using Student’s *t*-test compared to control condition and considering ^∗^*p* < 0.05 and ^∗∗^*p* < 0.01.

Supplementary Figure 5 BCL-2 family of proteins expression in BCP-ALL PDX cells after sunitinib treatment. Western blot analysis for anti-apoptotic and BIM proteins in BCP-ALL PDX cells after 16 h of treatment with sunitinib 100 nM and sunitinib 1,000 nM. Quantification of optical density for each protein was normalized to actin, and fold-change was calculated comparing to protein expression in the control condition.
