# Supplementary figures and images for: MCL-1 Inhibition Overcomes Anti-apoptotic Adaptation to Targeted Therapies in B-Cell Precursor Acute Lymphoblastic Leukemia
Source: Front Cell Dev Biol. 2021 Sep 9;9:695225. doi: 10.3389/fcell.2021.695225 (PMC8458912; doi:10.3389/fcell.2021.695225)

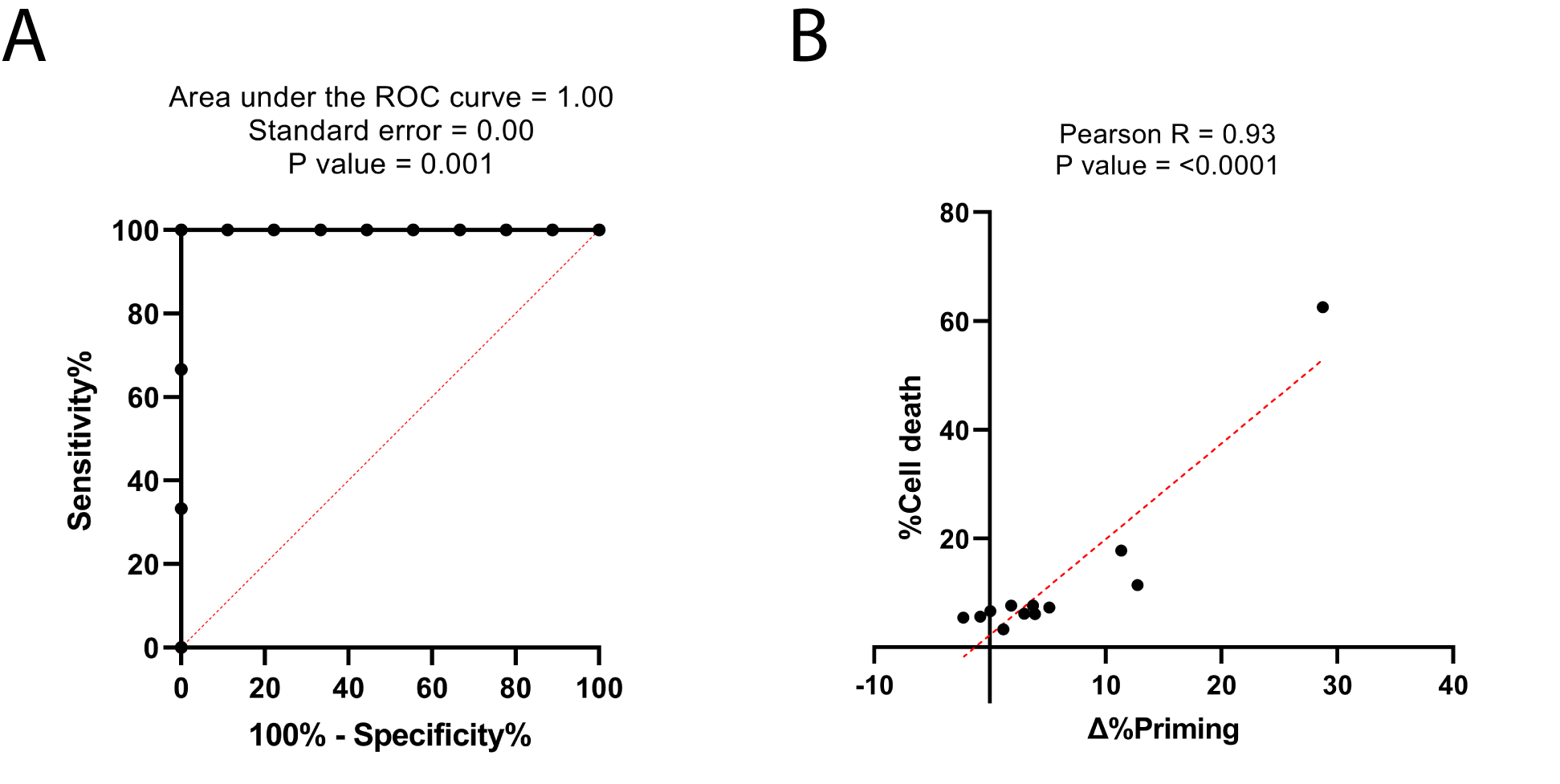

Supplement: Supplementary Figure 1 — (A) ROC curve analysis using the values of Δ%priming in NALM-6 and SEM cell lines establishing 10% as the cell death threshold for responders and non-responders. (B) Correlation between Δ%priming and %cell death analyses. [file Image_1.TIF]

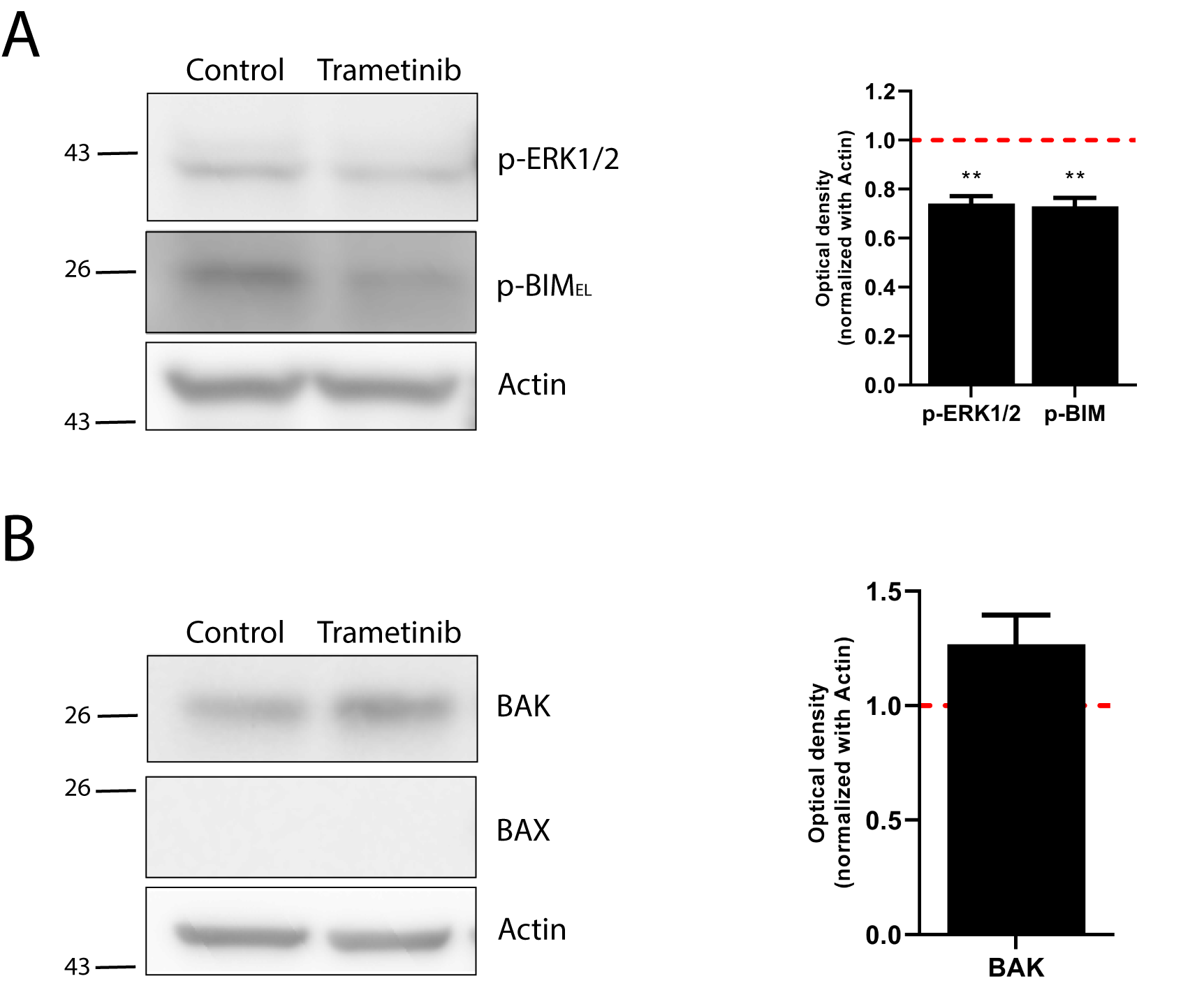

Supplement: Supplementary Figure 2 — (A) Western blot analysis of phospho-ERK1/2 and phospho-BIM in NALM-6 cell line after 16 h of treatment with trametinib 100 nM. (B) Western blot analysis of BAK and BAX in NALM-6 cell line after 16 h of treatment with trametinib 100 nM. Quantification of optical density for each protein was normalized to actin, and fold-change was calculated comparing to protein expression in the control condition. All results are expressed as the mean ± SEM of at least three biologically independent replicates. Statistical significance was calculated using Student’s t-test compared to control condition and considering ∗p < 0.05 and ∗∗p < 0.01. [file Image_2.TIF]

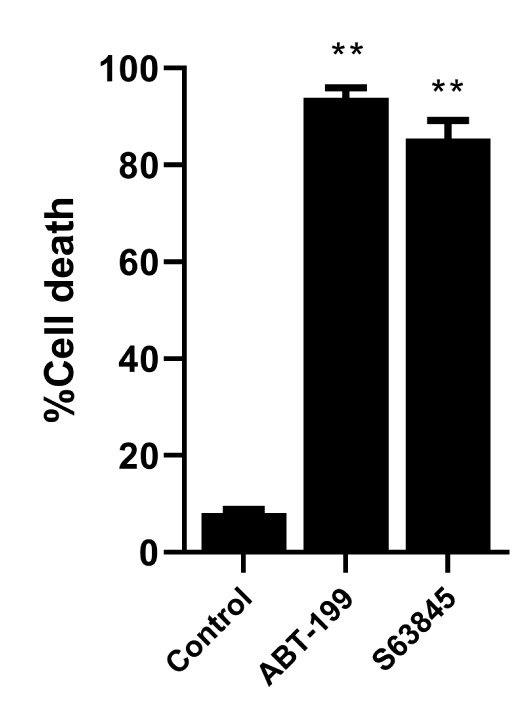

Supplement: Supplementary Figure 3 — ABT-199 and S63845 induce cytotoxicity in the SEM cell line. Cytotoxicity expressed as percentage of dead cells after 72 h of treatment with 100 nM ABT-199 and 1,000 nM S63845, as assessed by an Annexin V/DAPI staining. All results are expressed as the mean ± SEM of at least three biologically independent replicates. Statistical significance was calculated using Student’s t-test compared to control condition and considering ∗p < 0.05 and ∗∗p < 0.01. [file Image_3.TIF]

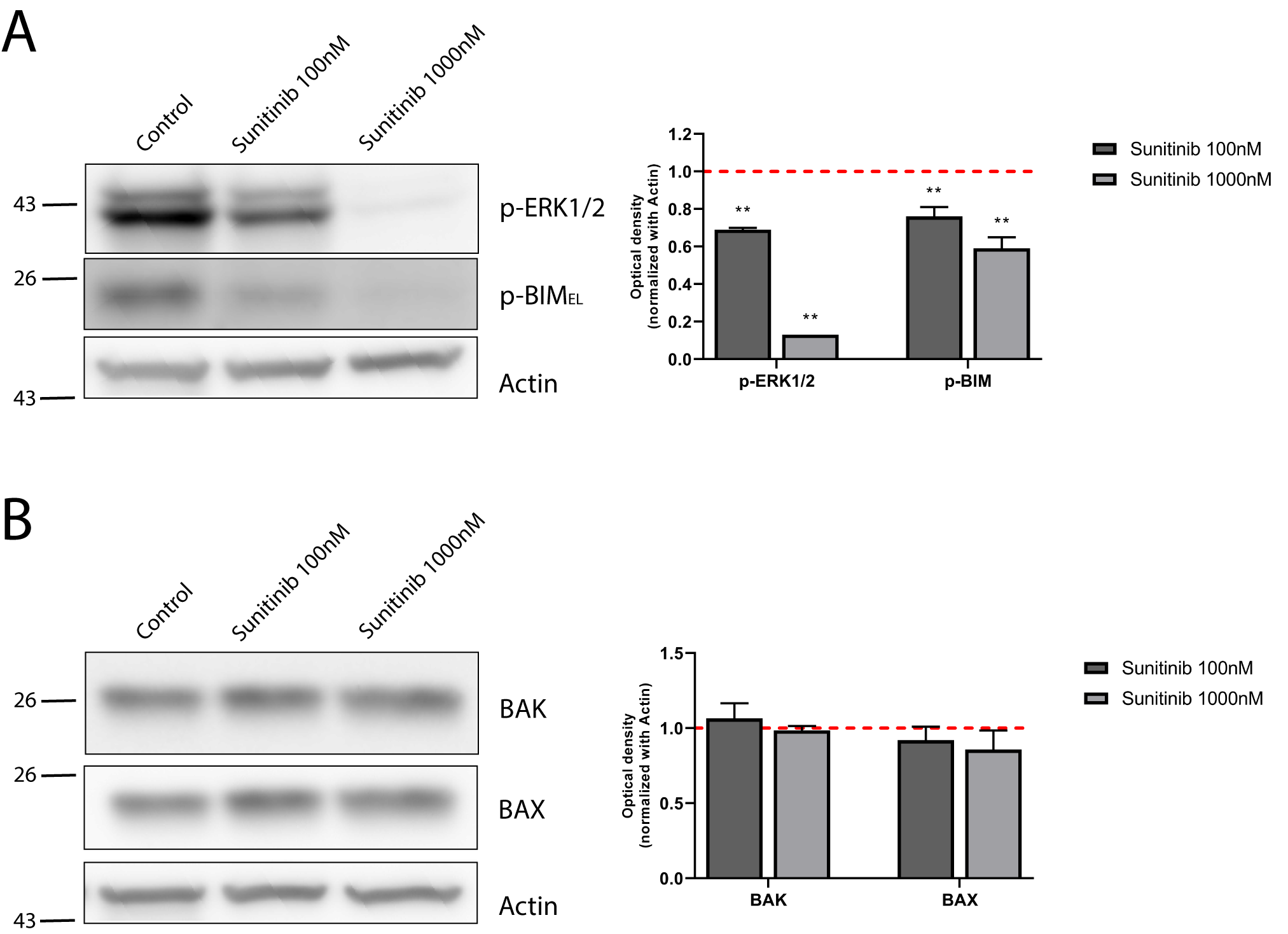

Supplement: Supplementary Figure 4 — (A) Western blot analysis of phospho-ERK1/2 and phospho-BIM in the SEM cell line after 16 h of treatment with sunitinib 100 nM and 1,000 nM. (B) Western blot analysis of BAK and BAX in SEM cell line after 16 h of treatment with sunitinib 100 nM and 1,000 nM. Quantification of optical density for each protein was normalized to actin, and fold-change was calculated comparing to protein expression in the control condition. All results are expressed as the mean ± SEM of at least three biologically independent replicates. Statistical significance was calculated using Student’s t-test compared to control condition and considering ∗p < 0.05 and ∗∗p < 0.01. [file Image_4.TIF]

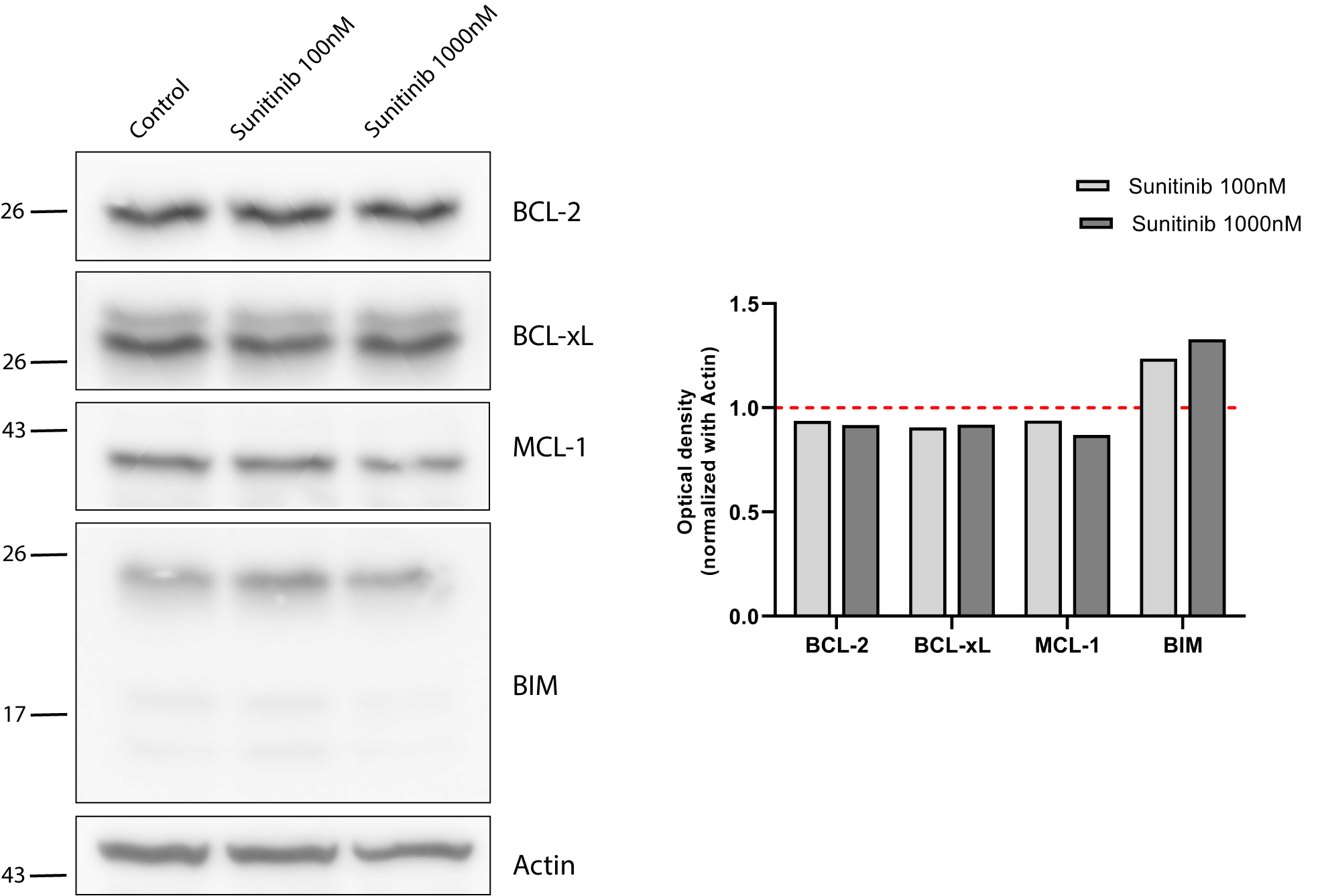

Supplement: Supplementary Figure 5 — BCL-2 family of proteins expression in BCP-ALL PDX cells after sunitinib treatment. Western blot analysis for anti-apoptotic and BIM proteins in BCP-ALL PDX cells after 16 h of treatment with sunitinib 100 nM and sunitinib 1,000 nM. Quantification of optical density for each protein was normalized to actin, and fold-change was calculated comparing to protein expression in the control condition. [file Image_5.TIF]
